# Development and evaluation of a direct disk diffusion, rapid antimicrobial susceptibility testing method from blood culture positive for Gram-negative bacilli using rapid molecular testing and microbiology laboratory automation

**DOI:** 10.1128/spectrum.02401-24

**Published:** 2025-05-15

**Authors:** Melvilí Cintrón, Brenden Clark, Edwin Miranda, Mauricio Delgado, N. Esther Babady

**Affiliations:** 1 Clinical Microbiology Service, Department of Pathology and Laboratory Medicine, Memorial Sloan Kettering Cancer Center 5803, New York, New York, USA; 2 Infectious Disease Service, Department of Medicine, Memorial Sloan Kettering Cancer Center 5803, New York, New York, USA; NHLS Tygerberg/Stellenbosch University, Cape Town, Western Cape, South Africa

**Keywords:** rapid antimicrobial susceptibility testing, *E. coli*, *Klebsiella pneumoniae *and* Pseudomonas aeruginosa*, EUCAST, laboratory automation, artificial intelligence

## Abstract

Rapid antimicrobial susceptibility testing (RAST) using disk diffusion (DD) from positive blood cultures (BC) can aid initiation of effective antimicrobial therapy. Rapid molecular blood culture identification panels (BCID) and microbiology laboratory automation (MLA) provide an opportunity to optimize RAST. This study aimed to evaluate and optimize RAST in combination with BCID and MLA for *Escherichia coli*, *Klebsiella pneumoniae,* and *Pseudomonas aeruginosa* positive BC. Monomicrobial BC positive for *E. coli*, *K. pneumoniae,* and *P. aeruginosa* by BCID between February 2020 and June 2021 were included. Images were captured by the MLA system after 4, 6, and 8 hours of incubation. The European Committee on Antimicrobial Susceptibility Testing (EUCAST) RAST breakpoints were used to interpret the zones of inhibitions measured by the WASPLab Halo Recognition software. Categorical agreement (CA) and error rates (minor [mE], major [ME] and very major [VME] errors) were calculated using automated microbroth dilution (aMBD) and DD as comparator methods. Ninety-one BCs met the inclusion criteria. CA between RAST and aMBD was >90% for most antimicrobials tested at all three time points for *E. coli* and *K. pneumoniae*. Overall, mE rates were <4%, ME rates ranged from 1% to 6% and VME rates ranged from 2% to 9%. Performance of RAST was lowest for piperacillin/tazobactam (71%–86%). For *P. aeruginosa,* CAs were mostly <90% with significant error rates. Combining rapid organism identification by BCID with automated RAST set-up and interpretation can facilitate the generation of early, preliminary antimicrobial susceptibility results for *E. coli* and *K. pneumoniae*.

**IMPORTANCE:**

Rapid antimicrobial susceptibility testing (RAST) for Gram-negative bacilli can quickly aid in the optimization of therapy for patients with bacteremia. In this study, a RAST workflow that includes identification using a molecular blood culture identification (BCID) panel, followed by manual set up and incubation on microbiology laboratory automation (MLA) with automated reading of zones of inhibition, was developed and evaluated. This workflow could provide AST results as soon as 6 hours after blood cultures flagged positive with categorical agreements > 90% for most antimicrobials tested. Additionally, the software used for automated measurements of the zones of inhibition provided accurate readings in 75% of the recorded measurements.

## INTRODUCTION

Due to the prevalence of high antibiotic resistance in Gram-negative bacilli, bacteremia caused by these organisms is associated with high mortality (1, 2). Consequently, obtaining rapid organism identification and antimicrobial susceptibility is critical to adequately treat patients and prevent the emergence of antimicrobial resistance. While identification of organisms can now be done in less than 2 hours with rapid multiplexed panels, traditional, phenotypic antimicrobial susceptibility testing (AST) still requires approximately 36–48 hours to generate results from the time a blood culture flags positive (3–8).

In 2019, the European Committee on Antimicrobial Susceptibility Testing (EUCAST) published a protocol for rapid antimicrobial susceptibility testing (RAST) directly from positive blood cultures using the disk diffusion (DD) method (9). The EUCAST RAST method provides guidance on the inoculum volume, quality control and breakpoints for interpretation after 4, 6, and 8 hours of incubation. Though the method is limited to certain antimicrobials and organism combinations, it can provide fast preliminary AST results. With the increased use of microbiology laboratory automation (MLA) and digital imaging in many microbiology laboratories (10–14), further opportunities exist to optimize the EUCAST RAST workflow.

The goal of this study was to develop and optimize a workflow for RAST DD for blood cultures positive with *Escherichia coli*, *Klebsiella pneumoniae*, and *Pseudomonas aeruginosa*.

## MATERIALS AND METHODS

### Study setting and design

**Fig 1:**
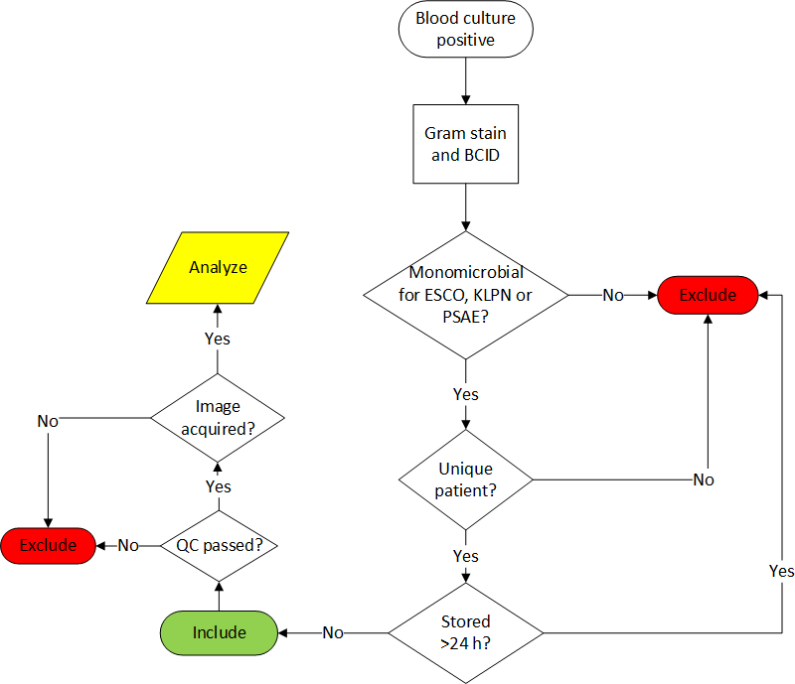
Inclusion criteria for modified RAST. BCID, blood culture identification panel; ESCO, *E. coli*; KLPN, *K. pneumoniae*; PSAE, *P. aeruginosa*; QC, quality control.

The study was performed at Memorial Sloan Kettering Cancer Center (MSK), a 514-bed tertiary cancer care center in New York City. The study period extended between February 2020 and June 2021. Positive blood cultures with Gram-negative bacilli identified as *E. coli*, *K. pneumoniae,* or *P. aeruginosa* by the Gram-negative ePlex Blood Culture Identification (BCID-GN) panel (Roche/GenMark, Carlsbad, CA) were included. Only the first positive blood culture (BC) bottle for each unique patient was included. Polymicrobial blood cultures, as determined by Gram stain and confirmed by the BCID or by culture, were excluded. Refer to inclusion criteria for further details (Fig. 1). All cultures were checked to confirm purity.

### Blood cultures and AST workflow

BC bottles were incubated in the BD BACTEC FX instrument (Becton Dickinson, Franklin Lakes, NJ) for up to 5 days. Positive BC bottles were aliquoted in 12 × 80 mm vacutainer tubes (Copan Diagnostics Inc, Murrieta, CA) and loaded on the WASPLab MLA system (Copan Diagnostics Inc, Murrieta, CA) to prepare the Gram stain slides and subculture to bacterial media per standard laboratory protocols. BCs positive for Gram-negative bacilli were immediately tested using the BCID-GN panel. The BCID-GN panel includes the following bacterial targets: *Acinetobacter baumannii, Bacteroides fragilis, Citrobacter* species*, Cronobacter sakazakii, Enterobacter* species (non-cloacae complex)*, Enterobacter cloacae* complex*, Escherichia coli, Fusobacterium nucleatum, F. necrophorum, Haemophilus influenzae, Klebsiella oxytoca, K. pneumoniae* group*, Morganella morganii, Neisseria meningitidis, Proteus* species*, Proteus mirabilis, Pseudomonas aeruginosa, Salmonella* species*, Serratia* species*, S. marcescens,* and *Stenotrophomonas maltophilia*. In addition to the bacterial targets, the following resistance genes are also included in the panel: CTX-M, IMP, KPC, NDM, OXA (OXA-23 and OXA-48), and VIM. AST was performed on isolated growth using the NM53 microbroth dilution panel in the MicroScan WalkAway system (Beckman Coulter, Brea, CA) as per manufacturer’s instructions, and interpretation was based on the instrument’s FDA breakpoints.

### Modified RAST method

RAST was performed following the EUCAST protocol with minor modifications (9). A 150 µL culture broth aliquot of BCID positive blood cultures for the organisms of interest was used as the inoculum. The inoculum was manually and evenly distributed over each of two 100 mm Mueller-Hinton (MH) agar plates (BD BBL, Franklin Lakes, NJ) using a Copan plastic SP 33 MM sterile swab (Copan Diagnostics Inc, Murrieta, CA). A total of 12 antimicrobial disks (BD BBL, Franklin Lakes, NJ) were dispensed but only results for amikacin (30 µg), ceftazidime (30 µg), ciprofloxacin (5 µg), gentamicin (10 µg), meropenem (10 µg), trimethoprim/sulfamethoxazole (23.75/1.25 µg), and piperacillin/tazobactam (100/10 µg) were analyzed. Except for ceftazidime (30 µg vs 10 µg) and piperacillin/tazobactam (100/10 µg vs 30/6 µg), the concentration of the disks used aligned with the EUCAST recommended concentrations. The DD plates were set up once identification was confirmed by the BCID-GN panel to be *E. coli*, *K. pneumoniae,* or *P. aeruginosa*, either the same day of cultures turning positive or from blood culture aliquots stored for less than 24 hours at 4°C. The 24 hour aliquots were brought to room temperature prior to setting up the DD plates. QC was performed each day of testing following the EUCAST RAST method recommendations.

### WASPLab and Halo software

**Fig 2:**
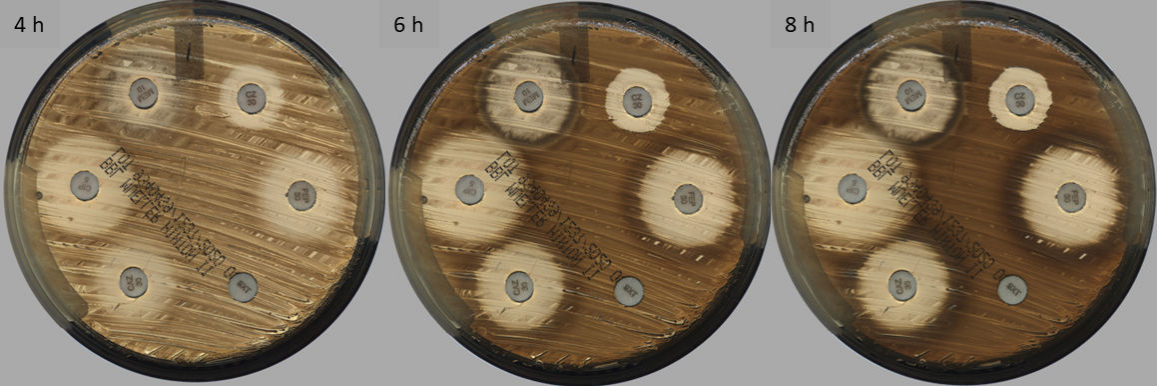
Example of confluence for *E. coli* rapid antimicrobial susceptibility testing after 4 hour, 6 hour, and 8 hour, respectively, incubation time.

All DD plates, QC, and patient samples were incubated in the WASPLab at 35°C, 5.5% CO_2_, laboratory standard incubation conditions. Images were taken at 4, 6, and 8 hours by the WASPLab Digital Imaging System and measurements of the zone diameters were captured by the WASPLab Halo Recognition software (Copan Diagnostics Inc, Murrieta, CA) at each defined time point only if confluent growth was observed (Fig. 2). All Halo measurements were downloaded from the WASPLab in .csv format and retrospectively verified against saved images for each reading time to confirm confluent growth on plates and defined zones of inhibition.

The accuracy of the Halo software measurements of the zone of inhibition was calculated as a percentage of all correct measurements over all measurements recorded. Measurements were deemed incorrect if Halo measurement did not agree with the standard reading of zones of inhibition as per the Clinical and Laboratory Standards Institute (CLSI) guidelines (e.g., not at edge of the defined zone of inhibition). Halo measurements were adjusted if deemed incorrect and median adjustment was calculated for each incubation time. An audit of adjusted measurements was done by selecting one culture per day of testing. Images were reviewed to determine the reason for the zone adjustment. In the case the zone was not recognized by the Halo software (e.g., image out of frame), the WASPLab manual measuring tool was used.

### Disk diffusion method

In addition to aMBD, standardized DDs were performed for the pure isolates included in the study. Briefly, 0.5 McFarland of each isolate was used to inoculate MH plates (BD BBL, Franklin Lakes, NJ) uniformly using a cotton swab. DD plates were incubated overnight at 35°C, ambient air. Zone diameters were measured manually and interpreted using the CLSI M100 Ed 34 breakpoints.

### Data analysis

Each zone diameter reading was rounded up (e.g., 14.6 = 15 mm) if applicable and interpreted in R studio version 4.05 as resistant (R), susceptible (S), or area of technical uncertainty (ATU) using the updated (15) (version 7.2) breakpoints at 4-, 6-, and 8-hour incubation. For the 8 hour time point, data were additionally interpreted using the short incubation CLSI breakpoints for the 8–10 hour (CLSI M100 Ed 34).

Categorical agreement (CA) was calculated using aMBD results for each of isolates as the comparison method. Minor errors (mE), major errors (ME), and very major errors (VME) were calculated as defined in the CLSI M52-ED1 (16). Only DD plates with confluent growth and defined zone diameters were read and categorized as S or R. Zones interpreted as ATU were excluded from error calculations as indicated (17). Performance of the modified RAST for each antimicrobial at each time point was considered acceptable as per CLSI guidelines: if CA was >90% with mE <10%, and VME and ME rates at ≤3% as. CA and error rates (mE, ME, and VME) were calculated in R studio version 4.05.

## RESULTS

During the study period, a total of 2,851 blood cultures flagged positive with Gram-negative bacilli with 346 *E. coli,* 333 *K. pneumoniae,* and 144 *P. aeruginosa* identified by the BCID-GN. A total of 726 cultures did not meet the inclusion criteria (e.g., no BCID, same patient, polymicrobial culture, QC failure, or storage >24 hours, etc.) for a final cohort of 91 positive blood cultures (36 *E. coli,* 34 *K. pneumoniae,* and 21 *P. aeruginosa*) from unique patients. aMBD results were reported at approximately 48 hours after the blood cultures flagged positive. These results were compared to those obtained by the modified RAST method at 4, 6, and 8 hours. All direct blood DDs were confirmed to have confluent growth at all time points (Fig. 1) and QC to be within the acceptable range for the antimicrobials and time points analyzed.

CA between RAST and aMBD for *E. coli* and *K. pneumoniae* was greater than 90% for almost all antimicrobials at all reading times, except for ciprofloxacin at 4 hours and piperacillin/tazobactam at all reading times (Table 1). The overall CA was 93% at 4 hours and increased to 97% at 8 hours. While the total number of mEs and VMEs were consistent at all reading times, MEs decreased as a function of time (i.e., 20 at 4 hours, 14 at 6 hours, and 7 at 8 hours). For *E. coli*, most CAs at 4 hours between RAST and aMBD were ≥93% except for piperacillin/tazobactam (67%) (Table 2). Only 1 (3%) mE, for ciprofloxacin, was observed at 4 hours, but a total of 4 MEs and 4 VMEs were observed for other antimicrobials. For *K. pneumoniae*, CA at 4 hours was ≥95% except for ciprofloxacin (75%), piperacillin/tazobactam (79%), and meropenem (85%). Most MEs were observed with ciprofloxacin (*n* = 7) and mEs were only observed for piperacillin/tazobactam (*n* = 2) (Table 3). Overall, 15 MEs were observed at 4 hours among four antimicrobials, but no VMEs were observed at this time point.

**TABLE 1:** *E. coli* and *K. pneumoniae* modified RAST combined results compared to aMBD^*a*^

	RAST interpretations^*b*^			aMBD interpretations^*c*^			Error rates				
Reading time	S	ATU	R	S	I	R	CA (%)	mE (%)	ME (%)	VME (%)	Total^*d*^
Amikacin											
4 h	39	31	^*e*^-	39	-	-	39 (100%)	-	-	-	39
6 h	66	4	-	66	-	-	66 (100%)	-	-	-	66
8 h	68	2	-	68	-	-	68 (100%)	-	-	-	68
Ceftazidime											
4 h	55	7	8	54	-	9	60 (95%)	-	1 (2%)	2 (22%)	63
6 h	56	5	9	54	-	11	63 (97%)	-	-	1 (18%)	65
8 h	56	3	11	55	-	12	66 (99%)	-	-	1 (8%)	67
Ciprofloxacin											
4 h	38	8	24	46	1	15	53 (85%)	1 (2%)	8 (17%)	-	62
6 h	45	4	21	50	1	15	60 (91%)	1 (2%)	5 (10%)	-	66
8 h	46	8	16	46	1	15	61 (98%)	1 (2%)	-	-	62
Gentamicin											
4 h	40	22	8	39	-	9	47 (98%)	-	-	1 (11%)	48
6 h	59	3	8	58	-	9	66 (99%)	-	-	1 (11%)	67
8 h	61	1	8	60	-	9	68 (99%)	-	-	1 (11%)	69
Meropenem											
4 h	50	15	5	55	-	-	50 (91%)	-	5 (9%)	-	55
6 h	57	8	5	62	-	-	57 (92%)	-	5 (8%)	-	62
8 h	59	9	2	61	-	-	59 (97%)	-	2 (3%)	-	61
Trimethoprim-sulfamethoxazole											
4 h	50	2	20	47	-	21	67 (99%)	-	-	1 (5%)	68
6 h	57	-	20	48	-	22	68 (97%)	-	-	2 (9%)	70
8 h	59	-	20	48	-	22	68 (97%)	-	-	2 (9%)	70
Piperacillin-tazobactam											
4 h	24	37	9	30	2	1	25 (76%)	2 (6%)	6 (20%)	-	33
6 h	36	27	7	40	2	1	37 (86%)	2 (5%)	4 (10%)	-	43
8 h	42	20	8	47	2	1	43 (86%)	2 (4%)	5 (11%)	-	50
Overall											
4 h	294	122	74	310	3	55	341 (86%)	3 (1%)	20 (6%)	4 (7%)	368
6 h	369	51	70	378	3	58	417 (95%)	3 (1%)	14 (4%)	5 (9%)	439
8 h	382	43	65	385	3	59	433 (95%)	3 (1%)	7 (2%)	4 (7%)	447

^*a*^ S, susceptible; I, intermediate; R, resistant; CA, categorical agreement; mE, minor error; ME, major error; VME, very major error; ATU, area of technical uncertainty; aMBD, automated microbroth dilution; RAST, rapid antimicrobial susceptibility testing; EUCAST, European Committee on Antimicrobial Ssusceptibility Testing.

^*b*^ EUCAST RAST breakpoints were used for interpretation.

^*c*^ aMDB panels were incubated and read at standard incubation times as per manufacturer’s instructions.

^*d*^ Total refers to the number of interpretable RAST results after the ATUs were removed.

^*e*^ - indicates zero.

**TABLE 2:** Comparison of *E. coli* modified RAST results to aMBD results^*a*^

	RAST interpretations^*b*^			aMBD interpretations^*c*^			Error rates				
Reading time	S	ATU	R	S	I	R	CA (%)	mE (%)	ME (%)	VME (%)	Total^*d*^
Amikacin											
4 h	18	18	^*e*^-	18	-	-	18 (100%)	-	-	-	18
6 h	34	2	-	34	-	-	34 (100%)	-	-	-	34
8 h	34	2	-	34	-	-	34 (100%)	-	-	-	34
Ceftazidime											
4 h	26	6	4	24	-	6	28 (93%)	-	-	2 (33%)	30
6 h	26	4	6	24	-	8	30 (94%)	-	-	2 (25%)	32
8 h	25	3	8	24	-	9	32 (97%)	-	-	1 (11%)	33
Ciprofloxacin											
4 h	20	2	14	21	1	12	32 (94%)	1 (3%)	1 (5%)	-	34
6 h	19	4	13	19	1	12	35 (97%)	1 (3%)	-	-	36
8 h	23	-	13	23	1	12	35 (97%)	1 (3%)	-	-	36
Gentamicin											
4 h	22	7	7	21	-	8	28 (97%)	-	-	1 (13%)	29
6 h	29	-	7	28	-	8	35 (97%)	-	-	1 (13%)	36
8 h	28	1	7	27	-	8	34 (97%)	-	-	1 (13%)	35
Meropenem											
4 h	18	7	1	19	-	-	18 (97%)	-	1 (5%)	-	19
6 h	34	2	-	34	-	-	34 (94%)	-	-	-	34
8 h	34	2	-	34	-	-	34 (97%)	-	-	-	34
Trimethoprim-sulfamethoxazole											
4 h	18	2	16	17	-	17	33 (97%)	-	-	1 (6%)	34
6 h	20	-	16	18	-	18	34 (94%)	-	-	2 (11%)	36
8 h	19	-	17	18	-	18	35 (97%)	-	-	1 (6%)	36
Piperacillin-tazobactam											
4 h	6	27	3	9	-	-	6 (67%)	-	3 (33%)	-	9
6 h	13	22	1	14	-	-	12 (93%)	-	1 (7%)	-	14
8 h	17	15	4	21	-	-	17 (81%)	-	4 (19%)	-	21

^*a*^ S, susceptible; I, intermediate; R, resistant; CA, categorical agreement; mE, minor error; ME, major error; VME, very major error; ATU, area of technical uncertainty; aMBD, automated microbroth dilution; RAST, rapid antimicrobial susceptibility testing; EUCAST, European Committee on Antimicrobial Susceptibility Testing.

^*b*^ EUCAST RAST breakpoints were used for interpretation.

^*c*^ aMDB panels were incubated and read at standard incubation times as per manufacturer’s instructions.

^*d*^ Total refers to the number of interpretable RAST results after the ATUs were removed.

^*e*^ - indicates zero.

**TABLE 3:** Comparison of *K. pneumoniae* modified RAST results to aMBD results^*a*^

	RAST interpretations^*b*^			aMBD interpretations^*c*^			Error rates				
Reading time	S	ATU	R	S	I	R	CA (%)	mE (%)	ME (%)	VME (%)	Total^*d*^
Amikacin											
4 h	21	13	^*e*^-	21	-	-	21 (100%)	-	-	-	21
6 h	32	2	-	32	-	-	32 (100%)	-	-	-	32
8 h	34	-	-	34	-	-	34 (100%)	-	-	-	34
Ceftazidime											
4 h	29	1	4	30	-	3	32 (97%)	-	1 (3%)	-	33
6 h	30	1	3	30	-	3	33 (100%)	-	-	-	33
8 h	31	-	3	31	-	3	34 (100%)	-	-	-	34
Ciprofloxacin											
4 h	18	6	10	24	-	3	21 (75%)		7 (28%)	-	28
6 h	22	4	8	27	-	3	25 (83%)		5 (19%)	-	30
8 h	23	8	3	23	-	3	26 (100%)			-	26
Gentamicin											
4 h	18	15	1	18	-	1	19 (100%)	-	-	-	26
86 h	30	3	1	30	-	1	31 (100%)	-	-	-	31
8 h	33	-	2	33	-	1	34 (100%)	-	-	-	34
Meropenem											
4 h	22	8	4	26	-	-	22 (85%)	-	4 (15%)	-	26
6 h	23	6	5	28	-	-	23 (82%)	-	5 (18%)	-	28
8 h	25	7	2	27	-	-	25 (93%)	-	2 (7%)	-	27
Trimethoprim-sulfamethoxazole											
4 h	30	-	4	30	-	4	34 (100%)	-	-	-	34
6 h	30	-	4	30	-	4	34 (100%)	-	-	-	34
8 h	31	-	3	30	-	4	33 (97%)	-	-	1 (25%)	34
Piperacillin-tazobactam											
4 h	18	10	6	21	2	1	19 (79%)	2 (8%)	3 (14%)	-	24
6 h	23	5	6	26	2	1	24 (83%)	2 (7%)	3 (12%)	-	29
8 h	25	5	4	26	2	1	26 (90%)	2 (7%)	1 (4%)	-	29

^*a*^ S, susceptible; I, intermediate; R, resistant; CA, categorical agreement; mE, minor error; ME, major error; VME, very major error; ATU, area of technical uncertainty; aMBD, automated microbroth dilution; RAST, rapid antimicrobial susceptibility testing; EUCAST, European Committee on Antimicrobial Susceptibility Testing.

^*b*^ EUCAST RAST breakpoints were used for interpretation.

^*c*^ aMDB panels were incubated and read at standard incubation times as per manufacturer’s instructions.

^*d*^ Total refers to the number of interpretable RAST results after the ATUs were removed.

^*e*^ - indicates zero.

At 6 hours, the *E. coli* CA ranged from 93% to 100% with piperacillin/tazobactam having the lowest agreement (93%) (Table 2). As with the 4 hour reading, only 1 mE was observed and attributed to ciprofloxacin. One ME attributed to piperacillin/tazobactam was observed, while a total of 5 VMEs attributed to ceftazidime, gentamicin, and trimethoprim/sulfamethoxazole were observed. For *K. pneumoniae*, the CA was >90% except for ciprofloxacin (75%), piperacillin/tazobactam (79%), and meropenem (85%). Similar to the 4 hour time point, mEs were low with only two observed, all attributed to piperacillin/tazobactam, whereas the MEs decreased from 15 to 13 total, and no VMEs were observed (Table 3). For *P. aeruginosa*, CA could only be calculated for 3/5 antimicrobials evaluated as all results for ceftazidime and piperacillin/tazobactam fell within the ATU category (Table 4). CA was 75% for ciprofloxacin, 89% for meropenem, and 90% for amikacin.

**TABLE 4:** Comparison of *P. aeruginosa* modified RAST results to aMBD results^*a*^

	RAST interpretations^*b*^			aMBD interpretations^*c*^			Error rates				
Reading time	S	ATU	R	S	I	R	CA (%)	mE (%)	ME (%)	VME (%)	Total^*d*^
Amikacin											
6 h	19	1	1	19	1	^*e*^-	19 (95%)	1 (5%)	-	-	20
8 h	19	-	1	19	1	-	19 (95%)	1 (5%)	-	-	20
Ceftazidime											
6 h	-	14	-	-	-	-	-	-	-	-	-
8 h	-	16	-	-	-	-	-	-	-	-	-
Ciprofloxacin											
6 h	-	12	4	-	1	3	3 (75%)	1 (25%)	-	-	4
8 h	-	13	4	-	1	3	3 (75%)	1 (25%)	-	-	4
Meropenem											
6 h	18	1	-	16	2	-	16 (89%)	2 (11%)	-	-	18
8 h	18	1	-	16	2	-	16 (89%)	2 (11%)	-	-	18
Piperacillin-tazobactam											
6 h	-	17	-	-	-	-		-	-	-	-
8 h	-	17	-	-	-	-		-	-	-	-

^*a*^ S, susceptible; I, intermediate; R, resistant; CA, categorical agreement; mE, minor error; ME, major error; VME, very major error; ATU, area of technical uncertainty; aMBD, automated microbroth dilution; RAST, rapid antimicrobial susceptibility testing; EUCAST, European Committee on Antimicrobial Susceptibility Testing.

^*b*^ EUCAST RAST breakpoints were used for interpretation.

^*c*^ aMDB panels were incubated and read at standard incubation times as per manufacturer’s instructions.

^*d*^ Total refers to the number of interpretable RAST results after the ATUs were removed.

^*e*^ - indicates zero.

At 8 hours, CA for *E. coli* was ≥97% for all antimicrobials except for piperacillin/tazobactam (81%) (Table 2). A total of 1 mE, 4 MEs, and 3 VMEs were observed. For *K. pneumoniae,* CA ranged from 90% to 100% for all antimicrobials (Table 3). Consistent with the 4- and 6 hour time points, 2 mEs for piperacillin/tazobactam were observed. The overall MEs decreased as a function of time, with only 3 MEs at 8 hours. However, 1 VME was observed for trimethoprim/sulfamethoxazole (Table 3). For *P. aeruginosa*, the lowest CA was again observed with ciprofloxacin (75%) and highest CA with amikacin (95%) (Table 4). The 8 hour zones of inhibition collected in this study were also interpreted using the CLSI short incubation breakpoints for the 8–10 hours incubation. Only three antimicrobials are shared between the EUCAST and CLSI RAST methods: ceftazidime, ciprofloxacin, and meropenem. Ceftazidime was analyzed for *E. coli* and *K. pneumoniae*, while ciprofloxacin and meropenem included *P. aeruginosa*. Overall error rates increased when using these breakpoints regardless of the comparison method used (i.e., aMBD or DD) (Tables S1 and S2). CA was low, 48.5%–52.5%, with mEs being 33.2%–33.9%. The MEs ranged from 6% to 18.3%, with the highest rate being attributed to comparing the results to standardized DD.

To evaluate if errors were due to the comparison method, the RAST results were also compared to standardized DD from the pure isolates. Results were found to be similar to results using aMBD as the comparison method (Table S3 and S4). Overall CA for *E. coli* and *K. pneumoniae* was 93% vs 92% by aMBD at 4 hours, 94% at 6 hours with either method as comparison, and 94% vs 95% by aMBD at 8 hours (Table S3). The overall mE rates were a bit higher (3%–4% vs 1%) but still within acceptable criteria (<10%). However, MEs and VMEs decreased when using standardized DD as comparison. For *P. aeruginosa*, CA was similar at all time points, with the only difference observed being that with DD as comparison, mEs disappeared for amikacin, but MEs were observed (Table S4).

### WASPLab Halo recognition accuracy

At the 4 hour time point, a total of 480 readings were recorded by the software and 84 (17.5%) were adjusted to reflect the accurate measurement of the zone (Table 5). The zone adjustments had a median of 2 mm (range: 1–11 mm). At 6 hours, 148/565 (26.2%) readings were adjusted with a median adjustment of 2 mm (range: 1–18 mm) and at 8 hours a total of 156/561 (27.1%) were adjusted with a median adjustment of 2 mm (range: 1–19 mm). Overall, a total of 1,606 Halo measurements were recorded and 388 (24.2%) were adjusted. Adjustments were noted to increase as a function of time.

**TABLE 5:** Frequency of reading adjustments for zones measured by the WASPLab Halo recognition software^*a*^

Antimicrobial	4 h		6 h		8 h		Total	
	Modified (%)	*N*	Modified (%)	*N*	Modified (%)	*N*	Modified (%)	*N*
Amikacin	15 (22.1%)	68	26 (29.5%)	88	27 (31%)	87	68 (28.0%)	243
Ceftazidime	10 (14.5%)	69	21 (25.3%)	83	25 (30.1%)	83	56 (23.8%)	235
Ciprofloxacin	14 (20.3%)	69	26 (30.6%)	85	25 (29.8%)	84	65 (27.3%)	238
Gentamicin	11 (16.2%)	68	19 (27.9%)	68	21 (30.9%)	68	51 (25.0%)	204
Meropenem	15 (21.7%)	69	21 (23.9%)	88	23 (26.4%)	87	59 (24.2%)	244
Trimethoprim-sulfamethoxazole	8 (11.6%)	69	12 (17.4%)	69	17 (25.0%)	68	37 (18.0%)	206
Piperacillin-tazobactam	11 (16.2%)	68	23 (27.4%)	84	18 (21.4%)	84	52 (22.0%)	236
Total	84 (17.5%)	480	148 (26.2%)	565	156 (27.8%)	561	388 (24.2%)	1,606

^*a*^ *N,* total number of Halo measurements available.

An audit of 116/388 (29.9%) of the adjusted measurements revealed that most adjustments (106/116 [92.2%]) were due to the Halo software under calling the zone of inhibition (i.e., measurement not at the edge of the zone). Two less frequent adjustments were due to the disk being recognized but the zone of inhibition not being measured (9 [7.8%]), and in one instance (0.9%), there was an overcall of the zone. An example of the under call is shown in Fig. S1. Adjustments were independent of the organism, incubation time, and the antimicrobial analyzed.

## DISCUSSION

The aim of this study was to investigate the performance of a modified RAST DD method in combination with BCID and MLA for direct from blood AST results for *E. coli, K. pneumoniae,* and *P. aeruginosa*. The CAs and mE rates were within the acceptable criteria of greater than 90% and less than 3%, respectively, for most antimicrobial tested, but in some cases, the ME and VME rates were high. In the case of VMEs, the high rates were likely due to the low number of resistant isolates included. Overall, CA and error rates were independent of the method used as comparison (i.e., aMBD or standardized DD). Additionally, using updated breakpoints reduced the ATUs when compared to results using the breakpoints available at the time of the study (data not shown).

Results of this current study are in line with results of prior studies reporting high agreements between the EUCAST RAST method and standardized testing (9, 15, 17–30). A tendency to overcall resistance was observed for piperacillin/tazobactam as shown by the high number of MEs observed for this antimicrobial/organism combination, a trend also reported in other studies assessing the EUCAST RAST method (18, 25). The low CA observed for piperacillin/tazobactam results might be due to the difference in the formulation of the European disk (e.g., 30/6 µg) vs the concentration of the available disk in the United States and used in this study (100/10 µg). Differences could also be linked to the performance of the comparison methods themselves. For example, when compared to the gold standard, MicroScan has been reported to have high error rates for piperacillin/tazobactam which could be impacting the results of this study (31). Another possible contributing factor is the inherent differences between CLSI, EUCAST, and FDA breakpoints. Like other reports, most error rates decreased as a function of time (9, 15, 20, 27).

The workflow for RAST set up and reading can be streamlined by MLA, which has been reported by other groups (21, 23–25). MLA provides continuous media incubation which optimizes organism growth and, with the integration of digital imaging, provides the ability to take images at the exact incubation time for reading DDs. It also provides laboratories the ability to set up and read the RAST results throughout the day rather than having to batch these samples for set up and reading. However, this may not be possible in laboratories with limited operating schedules. As an example, a previous study using MLA for the EUCAST RAST method determined that to provide same-day results within their operational hours, only the 6 hour reading time was optimal (25).

The use of MLA also provides the opportunity to integrate artificial intelligence (AI) software in this process. The Halo software used in this study recognizes the position of each disk and provides a measurement of the zone of inhibition with the ability to adjust it if needed. This further simplifies the workflow as plates do not need to be removed from the incubators to be measured. A report of measurements can be generated along with the recorded images and used as an auditing tool to validate the reading made by the software. The Halo software was calculated to be highly accurate at reading the zone diameters with an overall adjustment rate of 24.2%. Most adjustments were due to the zone measurement not being at the edge of the zone of inhibition. These errors were random and expected to decrease as the AI is trained.

The current study has limitations. First, the number of isolates analyzed was relatively low due, in part, to the high number of isolates classified as ATUs particularly for *P. aeruginosa*. This contributed to the high error rates observed even though the absolute number of misclassified isolates was as low as 1. Finally, the EUCAST RAST method was modified to align with the laboratory’s operational workflow on the WASPLab MLA, which may have impacted the overall accuracy of the results obtained. However, QC was performed on each day of testing and assessed for acceptability.

In 2022, the CLSI AST Subcommittee published breakpoints for direct blood culture disk diffusion (DD) testing of several antimicrobial agents for Enterobacterales and *Pseudomonas aeruginosa*. The workflow and processes developed in the current study could be used to evaluate the CLSI method. In this study, the 8 hour data collected were also used to determine CA and error rates using the CLSI 8–10 hour breakpoints. As shown, overall error rates increased when using these breakpoints; however, a more thorough study needs to be performed as only the data for the three antimicrobials shared between the EUCAST and CLSI RAST methods were evaluated in this study.

To our knowledge, this study is the first to evaluate a modified RAST protocol for non-spiked *E. coli, K. pneumoniae,* and *P. aeruginosa* using a combination of BCID, WASPLab MLA, and the assessment of the accuracy of the Copan Halo software for measuring DD zones. Thus, this study further shows the benefit of including these emerging technologies to automate and integrate RAST methods and provide preliminary AST results.

## SUPPLEMENTAL MATERIAL

Figure S1 spectrum.02401-24-s0001.tif Example of Halo software reading error.

Supplemental tables spectrum.02401-24-s0002.docx Tables S1 to S4.

ASM does not own the copyrights to Supplemental Material that may be linked to, or accessed through, an article. The authors have granted ASM a non-exclusive, world-wide license to publish the Supplemental Material files. Please contact the corresponding author directly for reuse.
