# Supplementary figures and images for: Development and evaluation of a direct disk diffusion, rapid antimicrobial susceptibility testing method from blood culture positive for Gram-negative bacilli using rapid molecular testing and microbiology laboratory automation
Source: Microbiol Spectr. 2025 May 15;13(6):e02401-24. doi: 10.1128/spectrum.02401-24 (PMC12131842; doi:10.1128/spectrum.02401-24)

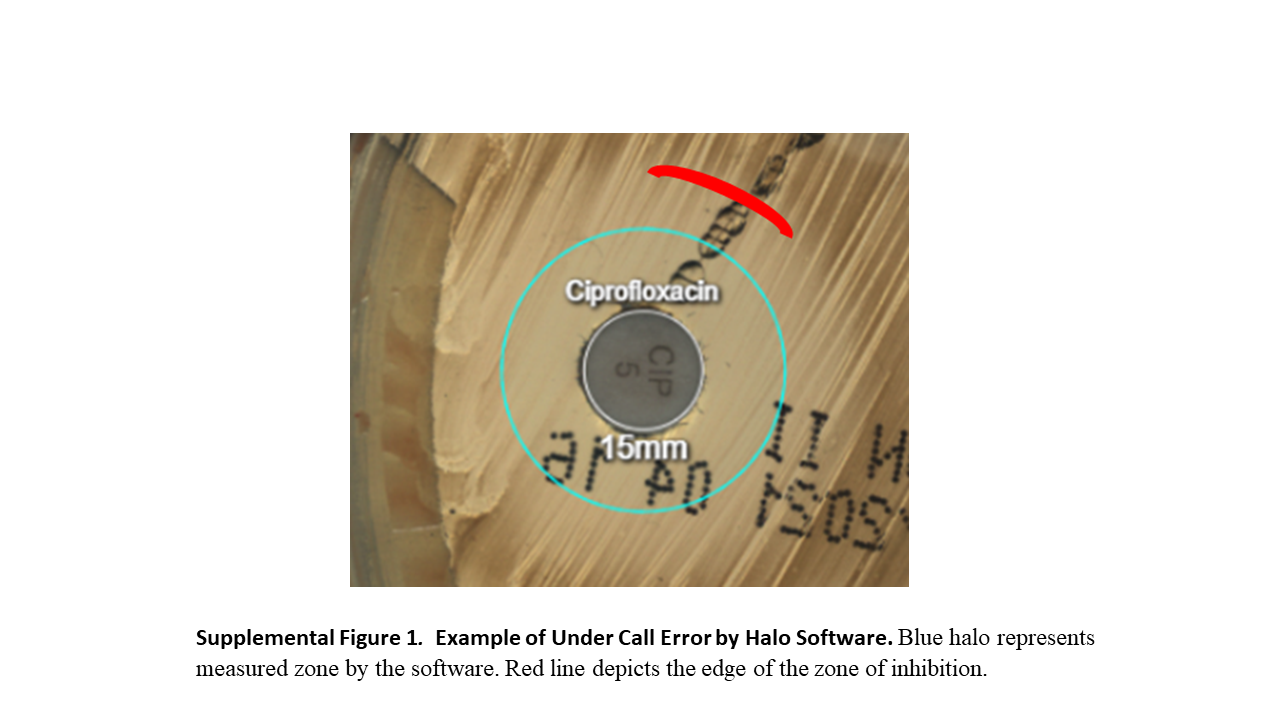

Supplement: Figure S1 — Example of Halo software reading error. [file spectrum.02401-24-s0001.tif]
