# Supplementary material for: Development and evaluation of a direct disk diffusion, rapid antimicrobial susceptibility testing method from blood culture positive for Gram-negative bacilli using rapid molecular testing and microbiology laboratory automation
Source: Microbiol Spectr. 2025 May 15;13(6):e02401-24. doi: 10.1128/spectrum.02401-24 (PMC12131842; doi:10.1128/spectrum.02401-24)
Supplement: Supplemental tables — Tables S1 to S4. [file spectrum.02401-24-s0002.docx]

**Supplemental Table 1*.* Combined Results for CLSI 8-10- hour Short Incubation Compared to aMBD**

**CLSI Interpretations^1^ aMBD Interpretations^2^  Error Rates**

| **Antimicrobial/Organism** | **S** | **I** | **R** | **S** | **I** | **R** | **CA (%)** | **mE (%)** | **ME (%)** | **VME (%)** | **Total** |
| --- | --- | --- | --- | --- | --- | --- | --- | --- | --- | --- | --- |

**Ceftazidime**

| *E. coli* | 9 | 16 | 10 | 24 | - | 11 | 19 | (54.3%) | 15 (42.9%) | - |  | - | 35 |
| --- | --- | --- | --- | --- | --- | --- | --- | --- | --- | --- | --- | --- | --- |
| *K. pneumoniae* | 17 | 11 | 6 | 31 | - | 3 | 20 | (58.8%) | 11 (32.4%) | 3(9.7%) |  | - | 34 |

**Ciprofloxacin**

| *E. coli* | 20 | 3 | 13 | 23 | 1 | 12 | 32 | (88.9%) |  | 4 (11.1%) |  | - | - | 36 |
| --- | --- | --- | --- | --- | --- | --- | --- | --- | --- | --- | --- | --- | --- | --- |
| *K. pneumoniae* | 15 | 12 | 7 | 31 | - | 3 | 18 | (52.9%) |  | 11(32.4%) |  | 3(9.7%) | - | 34 |
| *P. aeruginosa* | 9 | 3 | 4 | 11 | 1 | 4 | 11 | (68.8%) |  | 4 (25.0%) |  | - | 1(25.0%) | 16 |

**Meropenem**

| *E. coli* | 7 | 20 | 9 | 35 | - | 1 | 8 | (22.2%) | 25 (69.4%) |  | 3(8.6%) | - | 36 |
| --- | --- | --- | --- | --- | --- | --- | --- | --- | --- | --- | --- | --- | --- |
| *K. pneumoniae* | 5 | 8 | 21 | 34 | - | - | 5 | (14.7%) | 8 (23.5%) |  | 2(61.8%) | - | 34 |
| *P. aeruginosa* | 15 | 3 | 1 | 17 | 2 | - | 15 | (78.9%) | 3 (15.8%) |  | 1(5.9%) | - | 19 |

| **Overall** | 97 | 76 | 71 | 206 | 4 | 34 | 128 (52.5%) |  | 81 (33.2%) |  | 12 (6%) | 1(3%) | 244 |
| --- | --- | --- | --- | --- | --- | --- | --- | --- | --- | --- | --- | --- | --- |

*Note:* S= susceptible, I= Intermediate, R =resistant, CA= categorical agreement, mE = minor error, ME =major error, VME = very major error, CLSI= Clinical Laboratory Standards Institute. aMBD= automated Microbroth Dilution, ^1^ CLSI 8–10-hour short incubation breakpoints were used for interpretation. ^2^aMDB panels were incubated and read at standard incubation times as per manufacturer’s instructions.

**Supplemental Table 2*.* Combined Results for CLSI 8-10-hour Short Incubation Compared to DD**

**CLSI Interpretations^1^ DD Interpretations^2^ Error Rates**

| **Antimicrobial/Organism** | **S** | **I** | **R** | **S** | **I** | **R** | **CA (%)** | **mE (%)** | **ME (%)** | **VME (%)** | **Total** |
| --- | --- | --- | --- | --- | --- | --- | --- | --- | --- | --- | --- |

**Ceftazidime**

| *E. coli* | 9 | 16 | 10 | 27 | 3 | 5 | 14 | (40.0%) | 19 (54.3%) | - |  | - | 35 |
| --- | --- | --- | --- | --- | --- | --- | --- | --- | --- | --- | --- | --- | --- |
| *K. pneumoniae* | 17 | 10 | 5 | 30 | - | 2 | 19 | (59.4%) | 10 (31.3%) | 3(10.0%) |  | - | 32 |

**Ciprofloxacin**

| *E. coli* | 19 | 3 | 13 | 21 | 1 | 13 | 32 | (91.4%) |  | 2 (5.7%) |  | - | - | 35 |
| --- | --- | --- | --- | --- | --- | --- | --- | --- | --- | --- | --- | --- | --- | --- |
| *K. pneumoniae* | 14 | 11 | 8 | 26 | 2 | 4 | 16 | (50.0%) |  | 11(34.4%) |  | 4(15.4%) | 1 (25.0%) | 32 |
| *P. aeruginosa* | 9 | 3 | 4 | 10 | 1 | 5 | 10 | (62.5%) |  | 4 (15.4%) |  | - | 2 (40.0%) | 16 |

**Meropenem**

| *E. coli* | 7 | 19 | 9 | 35 | - | - | 7 | (20.0%) | 20 (57.1%) |  | 8(22.9%) | - | 35 |
| --- | --- | --- | --- | --- | --- | --- | --- | --- | --- | --- | --- | --- | --- |
| *K. pneumoniae* | 4 | 8 | 20 | 32 | - | - | 4 | (12.5%) | 8 (25.0%) |  | 20(62.5%) | - | 32 |
| *P. aeruginosa* | 15 | 3 | 1 | 16 | 3 | - | 12 | (63.2%) | 6 (31.6%) |  | 1(6.3%) | - | 19 |

| **Overall** | 94 | 73 | 70 | 197 | 10 | 29 | 114 (48.4%) |  | 80 (33.9%) |  | 36 (18.3%) | 3 (10.3%) | 236 |
| --- | --- | --- | --- | --- | --- | --- | --- | --- | --- | --- | --- | --- | --- |

*Note:* S= susceptible, I= Intermediate, R =resistant, CA= categorical agreement, mE = minor error, ME =major error, VME = very major error, DD= standardized disk diffusion, CLSI= Clinical Laboratory Standards Institute. ^1^CLSI 8-10-hour short incubation breakpoints were used for interpretation. ^2^CLSI breakpoints for standardized DD were used for interpretation.

**Supplemental Table 3*. E. coli* and *K. pneumoniae* Modified RAST Combined Results Compared to Standardized DD**

**RAST Interpretations^1^ DD Interpretations^2^ Error Rates**

| **Reading Time** | **S** | **ATU** | **R** | **S** | **I** | **R** | **CA (%)** | **mE (%)** | **ME (%)** | **VME (%)** | **Total*** |
| --- | --- | --- | --- | --- | --- | --- | --- | --- | --- | --- | --- |

**Amikacin**

| 4 h | 36 | 31 | - | 35 | 1 | - | 35 | (97%) | 1 (3%) |  | - | - | 36 |
| --- | --- | --- | --- | --- | --- | --- | --- | --- | --- | --- | --- | --- | --- |
| 6 h | 63 | 4 | - | 61 | 2 | - | 61 | (97%) | 2 (3%) |  | - | - | 63 |
| 8 h | 65 | 2 | - | 63 | 2 | - | 63 | (97%) | 2 (3%) |  | - | - | 65 |

**Ceftazidime**

| 4 h | 53 | 7 | 7 | 55 | 1 | 4 | 57 | (95%) | 1 (2%) | 2 (4%) |  | - | 60 |
| --- | --- | --- | --- | --- | --- | --- | --- | --- | --- | --- | --- | --- | --- |
| 6 h | 54 | 5 | 8 | 55 | 2 | 5 | 59 | (95%) | 2 (3%) | 1 (2%) |  | - | 62 |
| 8 h | 55 | 2 | 10 | 57 | 3 | 5 | 60 | (92%) | 3 (5%) | 2 (4%) |  | - | 65 |

**Ciprofloxacin**

| 4 h | 36 | 8 | 23 | 39 | 3 | 17 | 52 | (88%) | 3 | (5%) |  | 4 (10%) | - | 59 |
| --- | --- | --- | --- | --- | --- | --- | --- | --- | --- | --- | --- | --- | --- | --- |
| 6 h | 43 | 4 | 20 | 44 | 2 | 17 | 58 | (92%) | 2 | (3%) |  | 3 (7%) | - | 63 |
| 8 h | 44 | 7 | 16 | 41 | 2 | 17 | 57 | (92%) | 2 | (3%) |  | - | 1 (6%) | 60 |

**Gentamicin**

| 4 h | 37 | 22 | 8 | 37 | - | 8 | 45 | (100%) | - | - |  | - | 45 |
| --- | --- | --- | --- | --- | --- | --- | --- | --- | --- | --- | --- | --- | --- |
| 6 h | 56 | 3 | 8 | 56 | - | 8 | 64 | (100%) | - | - |  | - | 64 |
| 8 h | 58 | 1 | 8 | 58 | - | 8 | 66 | (100%) | - | - |  | - | 66 |

**Meropenem**

| 4 h | 47 | 15 | 5 | 52 | - | - | 47 | (90%) | - |  | 5(10%) | - | 52 |
| --- | --- | --- | --- | --- | --- | --- | --- | --- | --- | --- | --- | --- | --- |
| 6 h | 54 | 8 | 5 | 59 | - | - | 54 | (92%) | - |  | 5 (8%) | - | 59 |
| 8 h | 56 | 9 | 2 | 58 | - | - | 56 | (97%) | - |  | 2 (3%) | - | 58 |

**Trimethoprim-sulfamethoxazole**

| 4 h | 46 | 2 | 19 | 46 | - | 19 | 65 | (100%) | - | - |  | - | 65 |
| --- | --- | --- | --- | --- | --- | --- | --- | --- | --- | --- | --- | --- | --- |
| 6 h | 48 | - | 19 | 46 | - | 21 | 65 | (97%) | - | - |  | 2 (10%) | 67 |
| 8 h | 48 | - | 19 | 46 | - | 21 | 65 | (97%) | - | - |  | 2 (10%) | 67 |

**Piperacillin-tazobactam**

| 4 h | 24 | 36 | 7 | 22 | 6 | 3 | 22 | (71%) | 6 (19%) |  | 2 (9%) | 1 (33%) | 31 |
| --- | --- | --- | --- | --- | --- | --- | --- | --- | --- | --- | --- | --- | --- |
| 6 h | 35 | 27 | 5 | 29 | 8 | 3 | 30 | (75%) | 8 (20%) |  | 1 (3%) | 1 (33%) | 40 |
| 8 h | 41 | 20 | 6 | 34 | 9 | 4 | 37 | (79%) | 9 (19%) |  | - | 1 (25%) | 47 |

**Overall**

| 4 h | 279 | 121 | 69 | 286 | 11 | 51 | 323 | (93%) | 11 (3%) |  | 13 (5%) | 1 (2%) | 348 |
| --- | --- | --- | --- | --- | --- | --- | --- | --- | --- | --- | --- | --- | --- |
| 6 h | 353 | 51 | 65 | 350 | 14 | 54 | 391 | (94%) | 14(3%) |  | 10 (3%) | 3 (6%) | 418 |
| 8 h | 367 | 41 | 61 | 357 | 16 | 55 | 404 | (94%) | 16 (4%) |  | 4 (1%) | 4 (7%) | 428 |

*Note:* S= susceptible, I= Intermediate, R =resistant, CA= categorical agreement, mE = minor error, ME =major error, VME = very major error, ATU= Area of Technical Uncertainty, DD= standardized disk diffusion, RAST= Rapid Antimicrobial Susceptibility testing, CLSI= Clinical Laboratory Standards Institute. ^1^EUCAST RAST breakpoints were used for interpretation. ^2^CLSI breakpoints for standardized DD were used for interpretation.*Total refers to the number of interpretable RAST DDs after the ATUs were removed.

**Supplemental Table 4. Comparison of *Pseudomonas aeruginosa* Modified RAST Results to Standardized DD Results**

**RAST Interpretations^1^ DD Interpretations^2^ Error Rates**

| **Reading Time** | **S** | **ATU** | **R** | **S** | **I** | **R** | **CA (%)** | **mE (%)** | **ME (%)** | **VME (%)** | **Total*** |
| --- | --- | --- | --- | --- | --- | --- | --- | --- | --- | --- | --- |

**Amikacin**

| 6 h | 17 | 1 1 | 18 | - | - | 17 (94%) | - | 1 (6%) | - | 18 |
| --- | --- | --- | --- | --- | --- | --- | --- | --- | --- | --- |
| 8 h | 17 | - 1 | 18 | - | - | 17 (94%) | - | 1 (6%) | - | 18 |

**Ceftazidime**

| 6 h | - | 13 | - | - | - | - |  | - | - | - |  | - | - |
| --- | --- | --- | --- | --- | --- | --- | --- | --- | --- | --- | --- | --- | --- |
| 8 h | - | 15 | - | - | - | - |  | - | - | - |  | - | - |

**Ciprofloxacin**

| 6 h | - | 11 | 4 | - | 1 | 3 | 3 | (75%) | 1 | (25%) |  | - | - | 4 |
| --- | --- | --- | --- | --- | --- | --- | --- | --- | --- | --- | --- | --- | --- | --- |
| 8 h | - | 12 | 4 | - | 1 | 3 | 3 | (75%) | 1 | (25%) |  | - | - | 4 |

**Meropenem**

| 6 h | 17 | 1 | - | 14 | 3 | - |  | 14 (82%) | 3 (18%) |  | - | - | 17 |
| --- | --- | --- | --- | --- | --- | --- | --- | --- | --- | --- | --- | --- | --- |
| 8 h | 17 | 1 | - | 14 | 3 | - |  | 14 (82%) | 3 (18%) |  | - | - | 17 |

**Piperacillin-tazobactam**

| 6 h | - | 16 | - | - | - | - |  | - | - |  | - | - | - |
| --- | --- | --- | --- | --- | --- | --- | --- | --- | --- | --- | --- | --- | --- |
| 8 h | - | 16 | - | - | - | - |  | - | - |  | - | - | - |

*Note:* S= susceptible, I= Intermediate, R =resistant, CA= categorical agreement, mE = minor error, ME =major error, VME = very major error, ATU= Area of Technical Uncertainty, DD= standardized disk diffusion, RAST= Rapid Antimicrobial Susceptibility testing, CLSI= Clinical Laboratory Standards Institute. ^1^EUCAST RAST breakpoints were used for interpretation. ^2^CLSI breakpoints for standardized DD were used for interpretation.*Total refers to the number of interpretable RAST DDs after the ATUs were removed.
